# From Nutrition to Energy: Evaluating the Role of Rye (*Secale cereale* L.) Grain in Sustainable Food Systems and Biofuel Applications

**DOI:** 10.3390/foods14111971

**Published:** 2025-06-01

**Authors:** Adam Kleofas Berbeć, Marta Wyzińska

**Affiliations:** 1 Department of Agroecology and Economics, Institute of Soil Science and Plant Cultivation—State Research Institute, 24-100 Pulawy, Poland; 2 Department of Crops and Yield Quality, Institute of Soil Science and Plant Cultivation—State Research Institute, 24-100 Pulawy, Poland; mwyzinska@iung.pulawy.pl

**Keywords:** bioactive compounds, *Secale cereale*, grain quality, energy crops, diets, health

## Abstract

Rye (*Secale cereale* L.), a cereal with valuable agronomic and nutritional benefits, contributes to sustainable agriculture, especially in areas where more demanding crops cannot be cultivated due to the poor agronomic value of soil. This review explores rye grain quality optimization strategies through production techniques. The quality and yield of grain are under the significant impact of agronomic factors, such as variety selection, crop rotation, soil tillage, fertilization, sowing practices, chemical protection, and harvest timing. It is also under the strong influence of the chosen farm’s management strategy, like organic or conventional farming system. This review emphasizes its diverse potential utilization routes, and the importance of bioactive compounds, dietary fibers, phenolic acids, phytoestrogens, and benzoxazinoids that enhance its value as a functional food. Cereal grain with quality issues cannot be used as food for humans, however, it can still be utilized alternatively as a renewable biofuel. This review showed rye grain to have a potential to contribute to sustainable agriculture and at the same time build farms’ resilience through possible alternative utilization strategies. It can serve as both a food source and a sustainable biofuel, offering a dual-purpose solution within the circular bioeconomy.

## 1. Introduction

**Figure 1:**
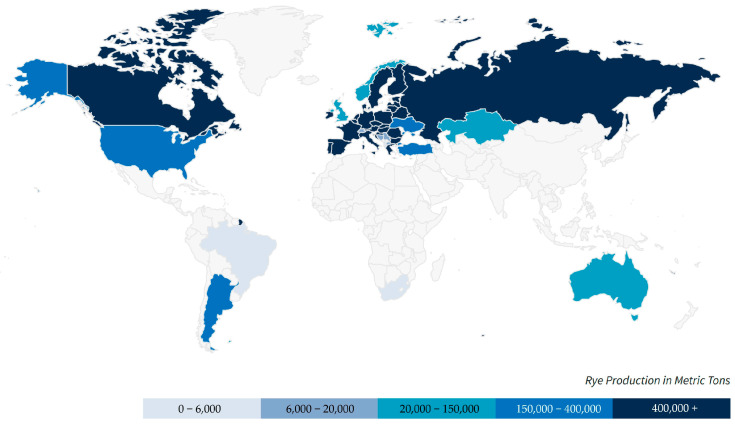
Global production of rye. Source: [4].

Rye (*Secale cereale* L.) has a long, widespread history of cultivation. It originated in around 6600 BCE as a weed species infesting mostly wheat and barley, and was domesticated in around 1800–1500 BCE. The first areas of cultivation were Asia, and Eastern and Central Europe. During the Middle Ages, it became a primary bread crop in multiple areas, particularly those characterized by poor soils and a cold climate [1,2,3]. According to USDA data [4], global production of rye was 10.6 million tons, with the majority of production located in the European Union (65%) [4] (Figure 1). Globally, in 2023, Germany was the largest producer of rye with 3.1 mln tons harvested, followed by Poland with 2.5 mln tons [5]. Globally, the production of rye grain is decreasing slightly (by approximately 1% annually). However, there are countries, such as Germany and Poland, where both the area of cultivation and production are increasing [4]. For example, in 2019, the area of land sown to rye was 904,000 hectares, representing a 179 thousand-hectare increase since 2015 [6].

Rye grain is mainly utilized as a food source (for rye flour production) and as a feedstock, but it is also used in industry, as a raw material for ethanol production. Around a third of global production is utilized to produce bread [7,8].

Rye can be cultivated on poor soils. Such soils, which are classified as light or very light, are common in Germany and Poland—the largest global producers of rye. For instance, sandy soils constitute more than 30% of the total arable area in Poland. These soils are characterized by their limited water storage capacity, low nutrient content, and typically low pH levels [8]. In such conditions, crop yields are usually low, particularly in years with high precipitation deficiencies. Rye, on the other hand, can produce relatively high yields on soil of poor quality, with yields even higher if it is cultivated on high-quality soils.

This paper aims to evaluate the quality attributes of rye grain in the context of its utilization in food products, highlight the challenges associated with the implementation of grain production technology, explore the potential impact on characteristics influencing its baking suitability, and also the alternative use opportunities.

## 2. Optimizing Grain Quality Through Production Technology

Rye can be successfully cultivated on soils of poor quality. It is the most stress-tolerant cereal, and is able to withstand conditions such as low pH and periodic moisture deficits better than other species. However, these stresses remain considerable for rye. Consequently, the average yield of rye grain in areas where it is cultivated can be relatively low, as cultivation conditions are often far from optimal. When cultivated on high-quality soils, rye can produce yields that are comparable with wheat, and hybrid forms have demonstrated the potential to surpass these yields. Recently, there has been a visible shift in the interest toward rye. This has resulted in rye being cultivated on higher-quality soils. This shift in interest is one result of market changes, with the price of rye grains for bread-making purposes reaching levels comparable with, or sometimes higher than, bread-making wheat [8,9]. Furthermore, the global rye market is projected to grow from USD 3.89 billion in 2023 to USD 5.83 billion in 2032 [10]. The factor most likely influencing rye’s market growth is health-conscious diets, resulting in the popularity of rye flour (and raw grains) in healthy, fiber-rich craft bakery products [11,12].

Several key quality parameters play a crucial role in determining the suitability of rye in flour production and bread-making. The most important of these are the initial and final glutination (gelatinization) temperatures and the maximum viscosity of the starchy paste. These have large impact on the dough behavior during mixing and baking, as well as the final texture of rye bread. Overall, the quality of rye flour is assessed based on a combination of starch behavior, enzymatic activity, and baking performance [13]. Some of these quality aspects are strongly influenced by rye cultivation conditions.

### 2.1. Selection of the Variety

Selecting a particular variety of rye grain for production can be crucial in determining its future quality. There are two main types of crop varieties: hybrids and open-pollinated (population) varieties. Hybrid varieties are created by crossing two parent lines to produce plants with improved traits like a higher yield or disease resistance (hetorosis effect is only visible in first F1 generation of plants). Open-pollinated varieties reproduce naturally, their seeds can be saved and replanted with consistent traits over generations. The genotype of the crop has a significant impact on its disease resistance, grain hardness, protein content, and falling number (an indicator of enzyme activity), as well as its tolerance to adverse weather conditions. It also determines whether the grain is utilized for food, feed, or industrial purposes [14,15,16]. The selection of variety to be the most suited for the local conditions allows for its rapid development and growth, but also its resistance to diseases—limiting the number of chemical treatments needed—which is of great importance to consumers seeking healthy food. The number of chemical treatments can influence both the rate of pesticide residues found in grain, but also the infestation of rye with diseases. In the case of rye, it is of particular importance to select varieties that exhibit an enhanced resistance to snow mold, brown rust, powdery mildew, and ergot. Even a minimal quantity of ergot (*Claviceps purpurea*) cones in the grain makes rye’s grain unsuitable for human consumption, as regulatory limits for ergot contamination are extremely strict. As an example of the importance of variety selection in disease resistance, Dańkowskie Złote has demonstrated a superior resistance to ergot among population varieties, making it a preferred choice in regions that are prone to high humidity or rainfall during the flowering phase [8,16,17].

The variety selected also impacts the grain morphology and nutritional profile, including the starch and fiber content and micronutrient levels. Some varieties are bred for a higher starch content (e.g., for distilleries), while others are bred for milling purposes (e.g., higher flour extraction rates, superior baking properties). Varieties with a higher fiber and micronutrient level can also be bred to meet the needs of a growing health-conscious market [18].

### 2.2. Crop Rotation

Crop rotation affects the quality of rye grain by influencing the availability of nutrients in the soil, as well as the pest and disease pressure and the soil’s structure (which is associated also with the soil’s water-holding capacity). In general, simplified, short crop rotations are common in field crop production [19]. This is due to the limited number of species that can be harvested and sold at a good price on the market. Wheat accounts for almost 50% of the cereal area of the EU, followed by barley and grain maize, each representing about 15% of the cereal area. Other cereals like rye, oats, and triticale are grown in smaller quantities [20]. The number of cultivable species in the lightest soils is particularly limited, which results in the frequent cultivation of rye after poor fore crops. This is because, in general, producers allocate the final position in the rotation to rye, which has a notable impact on the grain yield and quality. It is important to note, however, that the negative response of rye to cereal fore crops is less pronounced than that of wheat, barley, or triticale [21].

The life cycle of weeds, but also pests and diseases, is often directly linked to the life cycle of specific crop species. Diverse crop rotations are breaking the life cycle of pathogenic organisms. Conversely, simplified rotations are promoting the accumulation of pests and diseases in soil and plant residues. The spread of diseases can negatively impact the grain quality, e.g., through the development of fusarium and mycotoxin accumulation. Moreover, cereals with lower weed infestations require lower doses of herbicides, which reduces the risk of contamination of the grain with herbicide residues. Crops with a lower weed infestation also have better access to light, water, and nutrients, which directly impacts their quality [22].

More diverse crop rotations have a positive effect on the soil aggregate structure and increase the content of organic matter. These two soil properties are linked to the soil’s water and cation capacities. Better habitat conditions result in more uniform, fuller grains with a higher thousand-grain weight (WTG). This could be further supported by cultivating soil structure-forming crops, such as legumes and fodder crops. Rye grown after such crops often has a higher grain protein content (due to the increased mineral nitrogen availability in the soil). Diversified crop rotations also increase the availability of micronutrients, such as iron, manganese, and zinc, which is important for human and animal nutrition [23,24].

In practice, rye is often cultivated in succession (monoculture). The principal risk associated with monoculture rye crops is the proliferation of weeds resulting from canopy thinning and reduced plant competitiveness, as well as higher risks of fungal diseases [25].

### 2.3. Soil Tillage

The selection of the soil tillage strategy and its intensity have an impact on rye’s grain quality through a direct impact on the soil properties, including its fertility, but also influence crop development.

The general goal of soil tillage is to prepare the soil for crop sowing, emergence, and development. There are numerous methods that can be chosen to prepare soil for the cultivation of crops. Chosen methods impact the soil porosity and water-holding capacity, which impact crops’ root development. The introduction of agricultural systems where crop tillage is replaced with direct seeding (no-till systems), and where crop residues are left of field, result in an improved input of crop residues to soil, which leads to a higher soil organic matter content. This result improves the water availability in the arable soil layer and also make the soil more fertile with nutrients, which at the end impacts the grain quality [18,26].

On the other side, simplified tillage also brings challenges, of which one of the most significant is the increased prevalence of weed infestation. In a study by Parylak and Oliwa [27], shallowing of the ploughing depth (ploughing is the most basic tillage practice, with the plough working depth set at approximately 30 cm) or replacing it with other, less intensive techniques (eg., harrowing) resulted in an increase in the weed infestation of rye by over 30%. Another disadvantage of simplified tillage is the increased prevalence of stem base and root diseases, which some authors [28] have identified as a consequence of increased weed infestation. One consequence of the aforementioned diseases is lodging, which can result in grain overgrowth and a significant infestation of ears by Fusarium diseases. This, in turn, can lead to the presence of mycotoxins in grains. 

On the other hand, there are also research results that show that the reduction of tillage intensity by the adoption of simplified and no-till systems does not have a significant impact on grain quality of rye. This research found no difference in the protein content, nor the content of the nutrients in grain cultivated using simplified tillage methods under both conventional and organic farming conditions. At the same time, authors found that other factors, such as the fertilization strategy, can have a significant impact on the grain quality [29].

### 2.4. Fertilization

Soil fertilization, particularly with nitrogen, which is the basic yield-forming soil nutrient, plays a crucial role in determining the quality of rye grain (*Secale cereale* L.). The optimal dose and timing of fertilizer application affect the protein content, thousand-grain weight (TGW), grain uniformity, and the presence of antinutritional substances.

Increasing the nitrogen fertilization doses leads to an increase in the protein content of rye grain. At the same time, excessive fertilization can have an adverse effect on the thousand-kernel weight and grain uniformity. Studies have shown that nitrogen doses above 90 kg·ha^−1^ can reduce the thousand-grain weight and uniformity of the grain. Furthermore, higher nitrogen doses can increase the concentration levels of antinutritional substances, such as alkylresorcinols and pentosanes [30,31].

The content of antinutritional substances in rye grain, such as alkylresorcinols, pentosanes, and trypsin inhibitors, is affected by the level of nitrogen fertilization but also by the weather conditions during vegetation. Higher doses of nitrogen can increase the levels of these compounds, reducing the grain’s feed value. The content of these substances is also higher in years with more adverse weather conditions (droughts, low temperatures, spring frosts). However, proper fertilizer management can help to control the levels of these substances [31,32].

Producing quality rye grains requires optimal plant nutrition, encompassing the full range of nutrients throughout the growing season. In light soil conditions, this is often a challenge, as periodic water shortages prevent efficient nutrient absorption. The only way to limit the adverse effects of this phenomenon is to ensure optimal conditions for the growth of the root system in the autumn, particularly by implementing a balanced basic fertilization program with a particular focus on phosphorus, which is not easily translocated into the soil profile [33], making surface application ineffective.

In the case of food-grade wheat, nitrogen fertilization is of paramount importance with regard to grain quality, in order to achieve a sufficiently high protein content in the grains. In the case of bread-making rye, the objective of fertilization is not primarily to influence the protein content of the grains. Consequently, the literature on the nitrogen fertilization of rye is less extensive than that on wheat. The optimal nitrogen application rate for rye is relatively low (100–105 kg ha^−1^) [34], as high doses cannot be efficiently utilized in light soil conditions, primarily due to the moisture deficiency often observed in light, sandy soils. Those doses can be lowered if rye is cultivated after nitrogen-reach crops. For instance, a study by Liszewski [35] showed the optimal nitrogen rate for rye sown after legumes to be 25 kg ha^−1^. Moreover, combined nitrogen and sulfur fertilization can improve the quality of rye grain. Studies have shown that the application of 60–90 kg N·ha^−1^ combined with 40 kg S·ha^−1^ fertilization led to an increase in the protein content and improved the biometric characteristics of the grain. However, exceeding these doses may reduce the efficiency of nitrogen utilization by plants [36].

The recommended distribution method for the production of rye grains of optimal quality is analogous to wheat. The initial nitrogen application for rye may require the incorporation of straw into the soil before autumn sowing. Approximately 8 kg of nitrogen should be applied per ton of straw [8]. Nitrogen application in autumn may also be justified in fields where straw is not applied, as applying nitrogen at a rate of 40 kg ha^−1^ in autumn resulted in considerable increases in the rye grain yield. The impact of a late nitrogen application on rye grain quality is less pronounced than in wheat, where late nitrogen fertilization (spraying) is standard practice for improving grain quality parameters. Studies in rye showed that additional nitrogen fertilization at the amount of 30 kg ha^−1^ just before the heading stage of rye did not improve the quality parameters of rye’s grain [8].

Fertilization with other nutrients and micronutrients can also have an impact on rye’s grain quality. This impacts both the technological quality of grain, and its nutritive value. Phosphorus is crucial (especially in the first development stages of plants) for the development of the root system. This affects the crop’s ability to uptake water and other nutrients from the soil. Fertilization with this nutrient can also make the crop more resistant to environmental stresses, improving the quality traits of rye grain: thousand-kernel weight and the uniformity of grains [37,38,39]. Potassium is mostly responsible for plants’ water management, thus it has impacts on plants’ ability to withstand droughts. It is also crucial for protein and carbohydrates synthesis. Fertilization with potassium can positively impact grains’ protein content and technological value [40,41]. Micronutrients, including zinc (Zn), selenium (Se), and iron (Fe), are essential for the functioning of antioxidant enzymes in plants. Ensuring an adequate supply of these components through fertilization affects plant health and grain nutritional value. An adequate supply of these elements can increase their content in grain, which is important from the point of view of human and animal nutrition. Fertilization with iron, zinc, and other micronutrients contributes to biofortification—the process of increasing the nutritional value of crops by enhancing their content using agronomic practices (like fertilization) [42,43,44].

### 2.5. Sowing Date and Density

The sowing date determines rye’s development and yielding potential. Proper selection of sowing has an impact on crop development, as it affects the weather conditions of vegetation. Numerous studies confirm that optimizing the sowing date is an important agrotechnical factor that can determine the grain quality characteristics, such as thousand-kernel weight, total protein content, and falling number. The detrimental impact of delayed sowing on yield is due to the shortened growth period before winter, which impairs the development of the plants’ roots and above-ground parts before the cessation of growth in autumn. Such practices often result in reduced yields and weights of a thousand grains [45]. On the other hand, early sowing (usually from mid-September to mid-October) promotes better rooting of plants, a longer autumn vegetation period, and a higher biomass accumulation, which results in higher yields and improved grain parameters [46,47]. Research by Nye [48] conducted in the US showed that the optimal sowing date significantly improves the efficiency of the sowing density, which is reflected in the number of ears and the thousand kernel weight. Kraatz et al. [49] confirmed that sowing has to be done on time, as sowing dates that are both too early and too late can reduce the grain quality by increasing the risk of frost damage or incomplete grain ripening. The sowing date also affects the nitrogen uptake efficiency, which determines the grain protein content—a key indicator of grain quality [46,50].

The importance of sowing density for rye is not always fully appreciated. Consequently, rye is frequently sown at an excessively high density, a common error committed with regard to all cereal species. An excessively dense rye bed fosters the emergence of lodging and leaf and ear diseases, thereby necessitating an augmented level of protection. However, the efficacy of such measures may ultimately be constrained. It should be noted that the occurrence of lodging can be a direct cause of fusariosis in ears, which in turn can result in the production of mycotoxins. The presence of these toxins can render grains unsuitable for use as food or animal feed. A more detailed description of this issue will be provided later in this chapter.

The optimal sowing rate must be considered in terms of the trade-off between increasing the sowing density and the subsequent increase in the number of ears per unit area. However, increasing the density also exacerbates the competition between plants for light, water, and nutrients, which can have a negative impact on growth. Consequently, the grain yield per ear is significantly diminished, resulting in a reduction in the overall grain yield per unit area [51]. The selection of an appropriate sowing date should be adapted to the climatic and soil conditions of the growing region in order to maximize both the yield and quality of rye grain.

### 2.6. Chemical Protection

Use of chemical plant protection products (PPPs), including fungicides, insecticides, and herbicides, has a significant impact on rye’s grain quality. This impact can be both positive and negative, depending on the type of PPP used, date, and dose used, as well as depending on the local conditions.

Herbicides can effectively eliminate the competition for environmental resources from weeds, leading to higher rye yields and better grain quality. In general, rye has a superior competitive ability against weeds in comparison with other cereals [52]. However, this does not make the cultivation of rye, especially in conventional farming systems with short crop rotations, herbicide-free, as the tweed pressure in such systems is high [28]. The use of sulfonylurea herbicides can affect the grain quality parameters, such as protein content and thousand-grain weight [53]. On the other hand, the incorrect use of herbicides may lead to the damage of rye plants and their grains (due to herbicides’ phytotoxicity). Moreover, the presence of herbicide residues, such as glyphosate, in grain may raise serious safety concerns [54].

Fungicides are essential in controlling fungal diseases. Such diseases lead to reduced yields, but also grain contamination with mycotoxins, especially deoxynivalenol (DON). The use of chemical protection against fungal diseases is typically less intensive for rye than for wheat, even though rye exhibits similar negative reactions to diseases. Consequently, if the rye field is compact and the weather conditions are conducive to disease development, an appropriate spraying regimen must be implemented. The first fungicide treatment is usually applied at the 1–2 knee stage. The second treatment is administered at the end of the shooting phase, when ear formation begins. If the risk is lower, a single fungicide treatment at the flag leaf stage may be sufficient. Rye is infected by species of the genus Fusarium [55]. *Fusarium culmorum*, *Fusarium avenaceum,* and *Microdochium nivale* are considered to be the most harmful to rye [56,57]. These diseases primarily spread through contaminated grain, post-harvest residues, soil, and weeds. Therefore, in addition to the chemical methods, the implementation of appropriate agronomic measures is essential in the fight against the Fusarium diseases of rye.

Insecticides protect rye from being damaged by insects, like the aphids *Sitobion avenae* and *Rhopalosiphum padi.* Those are common cereal pests, which mainly damage crop leaves and ears leading to a decreased yield quantity and quality (thousand kernel weight). Aphids are also vectors of viruses, such as the barley yellow dwarf virus (BYDV), which causes a significant loss in the yield quantity and quality [58,59].

Effective, non-chemical protection of rye against lodging is based on good practice, such as appropriate variety selection, sowing density, and rational nitrogen fertilization. In locations with a high yield potential (above 5–6 t ha^−1^), the use of retardants is also recommended. It was found that the use of retardants can have a beneficial effect on rye grain yield even when lodging does not occur [17].

The use of chemical plant protection products in cereal crops, including rye, may lead to the presence of pesticide residues in grain. This raises concerns about food quality and safety. Therefore, it is essential to perform chemical protection in accordance with the integrated plant protection principle, selecting non-chemical measures as the priority, and regularly monitor pesticide residues [60,61].

### 2.7. Harvest Date

The harvest date is of great importance with regard to the quality of rye grain. The harvest date has an impact on the technological value, storage capacity, and suitability for further processing of rye’s grain. Both early and late harvesting can lead to a deterioration in the quality parameters of the raw material.

It is recommended that the harvest is done as soon as possible, however not before the grain has reached full maturity, as rye is prone to overgrowth and, in wet weather, can rapidly decline in quality, rendering it unsuitable for baking purposes. Harvesting at full maturity, at the optimal moisture content (14–16%), ensures high physicochemical quality, with a favorable starch and protein content and appropriate mechanical structure. Early harvest can result in a higher grain moisture content and lower test weight, while delayed harvesting increases the risk of kernel sprouting and fungal infections, thereby compromising the grain quality [62,63].

Additionally, quality deterioration may occur during the storage period. In accordance with the findings of Ryniecki and Szymański [64], the optimal moisture content for the storage of rye (as with other cereals) is 14% for a period of up to six months, and 13% for longer storage periods.

### 2.8. Organic Farming Practices

Organic farming—as a whole management system of the agricultural production strategy—utilizes different management practices that, in their essence, are more environmentally friendly than their conventional counterparts. Modern organic agriculture is becoming an important part of the agricultural food production sector. This is driven by the expectations of consumers for more healthy, environmentally friendly food production, backed by national and international policies and strategies. The organic farming system is supporting the sustainable development goals, but also the biodiversity conservation and soil health goals, which are also important European strategies reflected in the Sustainable Development Goals [65], EU Biodiversity Strategy for 2030 [66], and EU Soil Strategy for 2030 [67]. This means that organic farming systems are becoming a more important part of the EU’s economy. A dynamic growth of area utilized under organic farming principles is observed in the European Union. According to Eurostat, the total area under organic farming is continuously increasing, and in 2022, it was assessed at 16.9 mln hectares, which constituted to more than 10% of the UAA of the European Union [68]. In 10 years, the share of organic farming has increased by 7.4 mln hectares (78%) [69]. This trend shows the importance of organic farming as a foundation of the “Farm to Fork” strategy of the European Union, which aims at increasing the share of more healthy, sustainable food in the diet of the European population [70].

Rye is an important component of organic crop rotations. This crop can be utilized as a food crop, cover crop, and service crop aimed at improving soil quality parameters. Rye is known for being able to help in the management of pests and diseases in organic crop rotations, but it can also be utilized as a “green fertilizer” that can contribute to the soil organic matter content, especially on weak, sandy soils. It is also known as a valuable pre-crop. Its rapid development and ability to withstand low temperatures makes rye a perfect crop to cover the soil in vegetation during autumn and winter, to protect it from erosion and the loss of nutrients due to leaching [71]. What is even more important in organic farming conditions is that rye is highly competitive against weeds due to its allopathic compounds, such as benzoxazinoids, which inhibit weed seed germination, while its rapid canopy development shades out emerging weed seedlings, thereby reducing the weed pressure without chemical inputs [72].

Also, the quality of rye’s grain cultivated under organic farming conditions might be different than under the conventional farming system. This is, as mentioned in the previous sub-chapters, because the quality of the grain is determined by numerous factors, including nutrient availability, weed and disease pressure, as well as climate conditions. Organic farming aims at utilizing natural (organic) fertilization, often made with resources available within the farm (constituting to the circular economy principles), such as composts, animal manure, and “green manure” (crops that are cultivated only to be incorporated into the soil and contribute to its fertility). These sources release nitrogen more slowly compared with synthetic fertilizers, often resulting in lower total nitrogen availability during critical periods of grain development. Consequently, organically grown rye tends to have lower crude protein levels compared with conventionally grown rye, although the protein quality, in terms of the amino acid balance, may not be significantly inferior [73].

Pest and disease management of rye cultivated under organic farming conditions are based mostly on the diversification (complexity) of crop rotation, utilization of genetic diversification (use of resistant varieties and hybrid varieties), and timely performance of agricultural management practices. The inability to use industrial-made fungicides is associated with a higher risk of fungal pathogens, such as *Claviceps purpurea* (Fr.) Tul. The infestation of rye by this pathogen results in decreased quality (and safety) and thus market value of rye’s grains [74]. However, properly planned crop rotation, with crops dedicated to contributing to soil health, can greatly lower the risk of this disease spread. For example, Bertholdsson [75] found that late harvests can increase the risk of grain sprouting in the ear and fungal infections, which negatively affects its quality.

Another important factor that could potentially influence the quality of organic grain is its purity. Organic farmers have limited measures to conduct weed management, and thus, the risk of seed contamination by weed seeds is higher than in conventional crops. Depending on the species of the seeds, the contamination could result in decreased grain quality, but also increased safety issues for animal and human health. The seeds of some species contain toxic substances that might result in serious health issues if consumed. Species that are considered poisonous, whose seeds can cause health problems, have been deliberately controlled by farmers for centuries, which means that the risk of their occurrence in the field is low (their reproduction rate was very low, and thus their seeds did not feed the soil seed bank—the primary seed reservoir for wild species). In addition, further cleaning of the grain further reduces this risk, but still the risk of contamination is present. One of the most historically known and dangerous species is the corn cockle (*Agrostemma githago* L.), an annual weed that was once common in cereal crops over Europe. *Agrostemma githago* L. seeds contain triterpenoid saponins, including gitagin. This compound inflames the mucous membranes of the digestive tract and has hemolytic properties, which means that it can lead to the breakdown of red blood cells [76]. Even low doses of *Agrostemma githago* L. can cause symptoms of acute poisoning, such as nausea, vomiting, diarrhea, and in more severe cases, liver and kidney damage [77]. Despite the extreme decrease of the *Agrostemma githago* L. population, it can still sporadically be found in cereal crops, especially in organic farming systems where chemical plant protection is limited [78,79]. Rye, due to its high competitiveness abilities against weeds, can significantly decrease the risk of contamination of grain with weed seeds. A study conducted under moderate climate conditions showed the organic rye weed infestation rate to be up to 80–95% lower than infestation rate of other cereals grown under organic farming conditions [80].

Lastly, there is an ongoing discussion on whether organic farming practices could increase the risk of contamination of cereal grain with mycotoxigenic fungi. Due to the absence of chemical plant protection, organic cereals seem to be at a higher risk of fungal diseases and mycotoxin contamination compared with conventional cereals. However, the scientific research results seem to disprove this theory, as mycotoxin levels of organic and conventional cereals reported in scientific studies are most often at the same or a lower level than in conventional farming systems. This mainly concerns toxins produced by fungi of the *Fusarium* genus, such as deoxynivalenol (DON), zearalenone (ZEA), and T-2 and HT-2 toxins. The results seems to be more dependent on environmental and agronomic factors than on the management system itself [81,82,83].

## 3. Grain Quality and Bioactive Substances in Rye Grains

### 3.1. Grain Quality

Grain intended for human consumption should be of a high quality. Physical parameters of grain, such as the 1000-grain (kernel) weight and grain density expressed as hectoliter weight, and falling number (an indicator of alpha-amylase activity and sprouting damage), are commonly used basic indicators of grain quality. The weight of 1000 grains (WTG) reflects the degree of grain filling and maturity, which directly affects the milling yield and the quality of the final rye products. Grain density, measured as hectoliter weight (kg hl^−1^), is an easy-to-perform and practical indicator of the overall grain quality. These two characteristics are taken into account when assessing commercially traded grain requirements by agricultural, industrial, and milling industries. According to the authors of numerous studies [18,84,85], the hectoliter weight ranges from 62.6 to 76.1 g, while the WTG value ranges from 26 to 34 g [86,87,88].

One of the key parameters in assessing the quality of rye grain is also the falling number (FN). It reflects the activity of the α-amylase enzyme. A high activity of this enzyme, often caused by the pre-harvest germination of the grain (grain germination while still in the cereal ear), leads to a decrease in the falling number, which negatively affects the baking quality of the flour. The values of this parameter range from 93 to 340 s [8,89]. The falling number is strongly dependent on weather conditions during grain ripening. High humidity and rainfall can lead to pre-harvest germination, increasing α-amylase activity and reducing FN. Hybrid rye varieties show greater FN stability compared with population varieties, but are still susceptible to weather conditions [90].

### 3.2. Bioactive Substances in Rye Grains

Rye grains contain a plethora of chemical compounds that are conducive to the formation of a nutritionally balanced human diet. The most valuable of these are dietary fiber components, including pentosans, fructans, and β-glucans, is their water-soluble fractions [91]. In addition to the aforementioned compounds, rye grains contain relatively high amounts of readily assimilable proteins and vitamins, as well as phenolic acids (e.g., ferulic and caffeic acids) and phytoestrogens (e.g., secoisolariciresinol—SECO and matairesinol—MAT [92]. Recently, research on rye grain qualities determining its usefulness in the food industry has also focused on hydroxamic acids. The study by Fomsgaard et al. [93] identified the following compounds from this group in wholemeal flour: 2-benzoxazolin (BOA), 7-methoxy-2-benzoxazolin (MBOA), lactams such as 2-hydroxy-1,4-benzoxazolin-3-one (HBOA), and 2-hydroxy-7-methoxy-1,4-benzoxazin-3-one (HMBOA). Additionally, 2,4-dihydroxy-1,4-benzoxazin-3-one (DIBOA) and 2,4-dihydroxy-7-methoxy-1,4-benzoxazin-3-one (DIMBOA) and their respective glucosides were present. The quantity of these compounds present in grains is not considerable; however, given the relatively large quantity of bread consumed as part of a typical diet, the potential for their introduction into the body is significant. It would be sufficient to increase the consumption of dark rye bread, as the aforementioned compounds are primarily found in the fruit and seed coat.

A number of clinical studies have been conducted to confirm the possibility of the medical use of selected compounds from the benzoxazole group [94]. A comparison of the chemical structure of the compound MBOA and melatonin reveals a similarity to the hormone responsible for increasing the production of the anti-inflammatory factor IL-10 in the body [95]. In light of these findings, there has been a push to encourage the consumption of rye bread in numerous countries, particularly in Western Europe. Given the popularity of rye in Poland, it would be beneficial to consider implementing similar initiatives in this country. It would be prudent to conduct further research into the possibility of influencing the content of these compounds in bread. One potential avenue for exploration would be the preference of grains from selected rye varieties for the production of healthy food.

Bioactive substances are essential nutrients, as well as non-nutritive compounds that occur naturally in raw materials or their processing products, and affect the body’s metabolic and physiological functions. These compounds have been demonstrated to reduce the risk of numerous chronic non-communicable diseases, including cancer, arteriosclerosis, and type 2 diabetes [96]. The greatest concentrations of biologically active substances in cereal grains are found in the fruit and seed coat and aleurone cells. Consequently, milling processes result in a significant reduction in these substances in the final product, light flour. The flours in question are distinguished by a diminished content of dietary fiber (as evidenced in Table 1), particularly the soluble fraction. Additionally, they exhibit a reduced concentration of minerals (zinc, iron, selenium), vitamins (B6, folic acid), antioxidants (tocotrienols, ferulic acid), and phosphorus [97].

The US National Institutes of Health has defined bioactive substances as chemical substances that are not essential for meeting basic nutritional requirements but are responsible for changes in health status. These substances are formed by a number of physiological transformations, which are classified in the scientific literature as first-order (primary) and second-order (secondary) transformations. The former are metabolites found in every plant, which perform basic physiological functions. These include substances that provide energy, facilitate the synthesis of other substances, or serve as storage materials. Examples of these substances include simple sugars, starch, fats, chlorophyll, amino acids, proteins, and nucleic acids. In contrast, the latter are the product of a specialized metabolism and cannot be attributed to a primary function in plant life [108]. The list of biologically active ingredients found in food is extensive and continues to grow [109].

#### 3.2.1. Phenolic Acids

**Figure 2:**
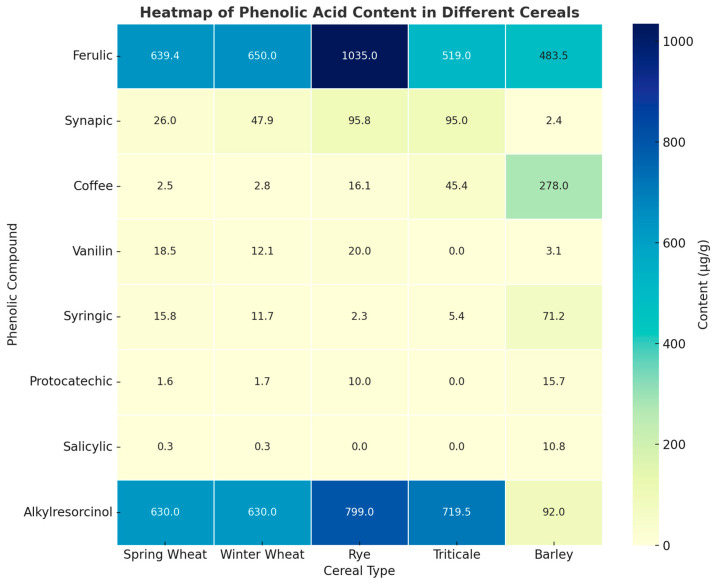
Heatmap of the phenolic acid content in different cereals. Source: [113,119,120,121,122,123,124,125,126,127,128,129,130,131,132,133,134,135,136].

**Figure 3:**
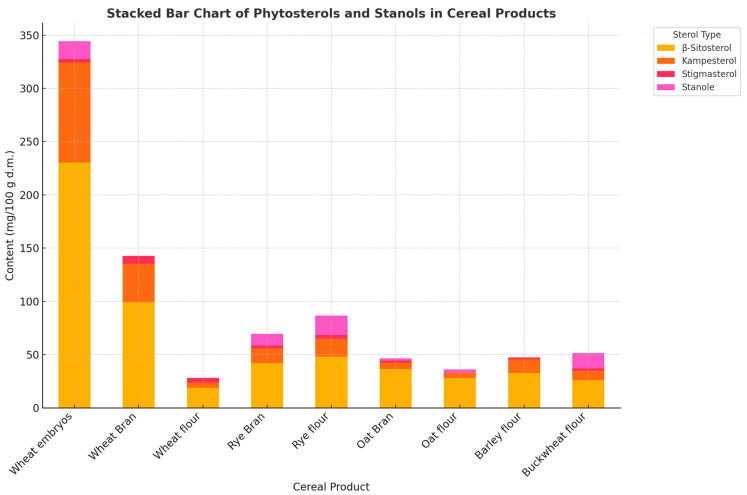
Content of total phytosterols and phytostanols in selected cereal products (mg 100g^−1^ d.m.). Source: [96,154,155,156].

Phenolic acids are present in the soluble free acid form and as soluble and insoluble esters [110]. Derivatives of benzoic and cinnamic acids are formed in the shiquinic acid pathway in plants. They contain a hydroxyl and a carboxyl group in their structure [97]. They occur in two forms, namely hydroxybenzoates and hydroxycinnamates. The most common hydroxybenzoates are p-hydroxybenzoic acid, vanillic acid, protocatechuic acid, and syringic acid, while the most common hydroxycinnamates are p-coumaric acid, ferulic acid, caffeic acid, and sinapinic acid. The concentration of free acids is typically low and varies depending on the species and the maturity of the plant. The total pool of phenolic acids in cereal grains is comprised of two distinct groups: phenylcarboxylic acids (p-hydroxybenzoic, salicylic, protocatechuic, vanillic, gallic, and ellagic) and phenylpropenoic acids (coffee, p-cumoric, ferulic, and synapinic). Collectively, these acids are referred to as phenolacids [111]. The most prevalent phenolic acid in cereal grains is trans-ferulic acid. As documented in the literature [112], grains of wheat, rye, barley, oats, and buckwheat are notable for their high concentration of phenolic acids. The highest concentrations of ferulic acid are found in wheat and rye grains, at 3.62 µg g^−1^ d.m. and 16 µg g^−1^ d.m., respectively. Vanillic acid is also present in notable quantities in rye, at 0.82 µg g^−1^ d.m. Additionally, p-coumaric acid is found in wheat. The lowest concentrations were observed in caffeic and sinapic acids, which were present in the smallest amounts [113,114,115]. The concentration of phenolic acids in cereal grains is largely contingent upon the specific variety (see Figure 2), the cultivation methodology employed, and the prevailing climatic conditions during the grain maturation phase [116]. The antioxidant mechanism of phenolic acids is contingent upon the number of hydroxyl groups present in the molecule. Cinnamic acid derivatives have been demonstrated to exhibit greater antioxidant efficacy than benzoic acid derivatives. These compounds are responsible for quenching radicals, protecting lipids from peroxidation, and also have the ability to chelate metal ions, catalyzing oxidation reactions. Phenolic compounds, through their properties, protect the human organism from oxidative stress and prevent the development of chronic non-infectious diseases, such as vascular atherosclerosis and cancer [117]. Figure 2 and Figure 3 present the content of phenolic acids in grain on different cereals. Lignians are a class of phenolic compounds that are present in plants. Some of these compounds are converted in the human body to lignans that are typical in mammals. They are distinguished by their phytoestrogenic activity. These properties may render them useful in the treatment of menopausal symptoms, cancer, or heart disease [97]. They are present in whole cereal grains. These substances are responsible for the formation of structural lignin blocks in cell walls. The primary sources of these compounds are cereal plants, although they are also present in flax seeds, fruits, and vegetables [118]. Additionally, these substances are present in plants as diglycosides, with two glucose residues attached to the OH group of the phenolic ring or side chains. As documented by Bingham et al. [118], urinary lignian levels demonstrate a notable increase following the consumption of a meal containing these substances.

#### 3.2.2. Dietary Fiber

Dietary fiber has the greatest influence on lipid and carbohydrate metabolism, and regulates the function of the entire gastrointestinal tract. This reduces the risk of atherosclerosis, ischemic heart disease, type 2 diabetes, and obesity. The consumption of dietary fiber has also been linked to a reduced risk of developing colorectal cancer. Pentosans, fructans, and β-glucans, particularly their water-soluble fractions, represent nutritionally valuable components of dietary fiber. The findings of the study conducted by Jasińska et al. [137] suggest that the highest concentration of dietary fiber components is present in the fruit and seed coat, as well as the aleurenic cell layer, while the lowest is observed in the inner tissues of the endosperm. They are primarily cell wall components [138]. Conversely, the research conducted by Nilsson et al. [139] suggests a notable correlation between the dietary fiber content and the bioactive component, specifically phytoestrogens, in rye grains.

#### 3.2.3. Tocols

Tocols constitute a group of compounds, namely vitamin E, which comprises eight congeners: four tocopherols (α-, β-, γ-, δ-) and four tocotrienols (α-, β-, γ-, δ-). These compounds are composed of 6-chromanol rings and a phytyl chain, which is saturated in the case of tocopherols and contains three double bonds in the case of tocotrienols. The dissimilarities in the structural configuration give rise to disparate biological activities among these substances. Tocopherols and tocotrienols are compounds with antioxidant properties that are found in cereal grains [97]. As evidenced in the literature [97,127], tocopherols and β-tocotrienols are predominantly located in the embryo, while tocotrienols are primarily distributed in the fruit and seed coat, with a presence in the endosperm.

#### 3.2.4. Phytoestrogens

Phytoestrogens are non-steroidal compounds that have estrogenic properties. The aforementioned compounds can be classified into three principal groups: isoflavonoids, lignans, and coumestans [140]. They have been demonstrated to possess antiviral, anticancer, bactericidal, and antifungal properties [141].

#### 3.2.5. Alkylresorcinols

Alkylresorcinols were first identified in the 1970s and 1980s as undesirable substances in feed grains, with evidence suggesting that they may even be harmful to monogastric animals. Currently, there is considerable interest in these compounds as a bioactive component of foodstuffs [125]. The alkylresorcinols present in cereal grains, particularly wheat and rye, have been the subject of extensive research and are among the most well-studied and well-known compounds in this field. The content of these substances in grains is largely dependent on the species of the cereal in question (see Figure 2). A review of the literature reveals a range of content in cereal grains. These substances are of plant origin [142]. They are derivatives of 1,3-dihydroxy-5-n-alkylbenzene, also known as orcin. Alkylresorcinols are essential for plant growth, adaptation, and defense against pathogens [125]. The first isolation of these compounds in cereal grains was reported by Wenkert [143] in wheat bran. In a subsequent study by Wierninga [144], the same substances were identified in rye grain. The defining characteristics of alkylresorcinols include the presence of an alkyl side chain comprising an odd number of carbon atoms in the aromatic ring at position 5 [142]. The aforementioned chain is typically saturated; however, unsaturated homologs or those containing an additional oxygen group also exist [64]. Alkylresorcinols are exclusively present in the outer layers of the granule [125]. Tłuścik et al. [145] observed that the embryonic part and endosperm are devoid of alkylresorcinols. The concentration of these compounds in rye grains exhibits considerable variability, with reported values ranging from 360 mg kg^−1^ to 2180 mg kg^−1^ [146,147]. This is notably higher than the levels observed in other cereal species.

The role of alkylresorcinols in biological activity and the regulation of physiological processes or metabolism has, until now, remained relatively unknown. It is established that they regulate cell growth processes, inhibit DNA and RNA synthesis, disrupt the enzymatic activity of proteins, interact with biological membranes, and regulate lipid oxidation processes. Furthermore, they have been demonstrated to possess antimutagenic, antibacterial, fungicidal, and cytotoxic properties. They are among the most effective substances in the prevention of cancer and ischemic heart disease. Furthermore, they delay the ageing process [125].

#### 3.2.6. Benzoxazinoids

Benzoxazinoids are commonly found among monocotyledonous species. They are secondary metabolites that have many important defensive and adaptive functions: bactericidal, fungicidal, and allelopathic, and they reduce the development and number of eggs of parasitic nematodes. They have been identified relatively recently in life and baked goods from this cereal species. Individual compounds from this group are characterized by pharmacological and health-promoting, as well as antimicrobial, anti-allergic, anti-inflammatory, or anti-cancer properties [97].

#### 3.2.7. Carotenoids

Carotenoids are compounds that give the yellow, orange, and red color to fruits, vegetables, and grains of some cereals. These compounds are characterized by valuable biological properties [148]. They are divided into two basic groups: carotenes, which contain 11 conjugated double bonds in the molecule; and xanthophylls, which contain oxygen in the form of carbonyl, epoxy, and hydroxyl groups in the molecule. Carotenoids are the main color substances of cereal grains. According to the literature [149], they are present in small amounts in all the anatomical parts of grains, mainly in the embryos. The main pigments of wheat include yellow lutein, as well as its mono- and diesters with fatty acids. According to Konopka et al. [149], the content of carotenoids in the starchy endosperm of spring wheat is ca. 3.5 mg kg^−1^, while in winter wheat, it is 2.4 mg kg^−1^. In rye grains, the carotenoid fraction consists of α-carotene, poly-cis-lycopene B, lutein, xanthophyll epoxides (5.6-epoxylutein), and taraxanthin, which is mainly located in the embryo and varies from 2.8 to 7.6 mg kg^−1^ [96]. In contrast, 5.6-epoxylutein and taraxanthin were found in oat grains [150].

#### 3.2.8. Flavonoids

Flavonoids are compounds that contain a diphenylpropane system made up of two benzoate rings in their molecule. They are commonly found in plants and differ in structure and properties. These compounds are characterized by many health-promoting pharmacological and biological properties. In the literature [117], authors point out anti-inflammatory, anti-allergic, anticoagulant, antiviral and anticancerogenic, antioxidant, diuretic, and detoxifying activities. These substances have the ability to modify the enzymes responsible for immune function, carcinogenesis, and cell transformation [96]. As reported by Rybka et al. [113], many flavonoids inhibit lipid preoxidation, improve vascular endothelial function, and inhibit platelet aggregation and muscle tone surrounding the arteries in vascular diseases. In cereal grains, these compounds are present in very small amounts and their presence has been confirmed mainly in the fruit and seed coat, aleurone cells, and husk (oats), but also in some cases in the embryo [150].

#### 3.2.9. Phytosterols and Phytostanols

Phytosterols are recognized as non-nutritive bioactive components with proven health-promoting properties, but also have known adverse effects on the human body. Sterols are included in a diverse group of plant secondary metabolites that are present in nuts, fruits, and seeds, among others [97]. Phytosterols are usually found in the free or esterified form. They are structural and functional analogs of cholesterol, synthesized by plants. They form part of the plant cell membranes and reduce the fluidity of their surface layer. They are 28- or 29-carbon polycyclic alcohols [151]. Sterols have a polycyclic system like cholesterol, with one hydroxyl group. The difference in their structure concerns the side chain. They may additionally contain one or two double bonds in this chain, and are richer in a methyl or ester group [152]. In their natural state, these compounds occur in the free form and as sterol or stanol esters of fatty acids, hydroxycinnamic acid, glucose, and glycolipids. Plant sterols have a safe status and health claims containing references to the beneficial effect of sterols on human health are accepted for various products by both the FDA and EFSA. According to the literature, only 15% of sterols are absorbed from the intestines, thus the active concentration in the body is low [97]. So far, almost 40 forms of plant phytosterols have been recognized, of which the best known and most common are: sitosterol and sitostanol, capesterol and campestanol, and stigmasterol, whose structure is most similar to the cholesterol ring [152]. Plant sterols are all the compounds included in this group, but in fact, sterols are unsaturated compounds, i.e., having double bonds in their ring, while stanols are their unsaturated forms, without double bonds. Quite commonly, phytosterols are found in plant products, but their amount is very small. The largest amounts are found in vegetable oils, legumes, sesame, sunflower, and other seeds. Trace amounts are found in vegetables, fruits, and whole grain cereals. Only 200–400 mg of phytosterols can be provided to the body through the diet. This is far too little to effectively reduce LDL cholesterol. Both sterols and stanols are poorly fat-soluble and water-insoluble [153]. The content of total phytosterols and phytostanols in selected cereal products is shown in Figure 3.

## 4. Alternative Use Scenario: Rye Grain Utilization as Biofuel

Rye grain can also be utilized outside of the food industry as a promising solid biofuel. The scientific evidence on rye’s grain HHV and its potential as solid biofuel is currently largely missing from the literature. However, rye’s unique agronomic benefits, environmental advantages, and energy potential reviled in some studies, shows this crop as a potential contributor to the sustainable development of the agricultural sector, while also contributing to farms’ resilience. Rye produces high yields in a wide range of, often challenging, soil and climatic conditions. Malone et al. [157] found the energy yields of rye to be up to 145.2 GJ per hectare. Such high values of energy yield makes rye a strong contender for biofuel use. Żabiński et al. [158], found rye’s heat of combustion at a level of 17.68 MJ kg^−1^, which made it a similar source of energy (in terms of calorific value) to wood pellets (usually 16–19 MJ kg^−1^).

On a chemical level, rye’s grain is well-suited for biofuel production. The most important primary element that contributes to biomass HHV is carbon (and, to a smaller extent, hydrogen). Carbon-rich compounds of biomass are cellulose, hemicellulose, and lignin. In cereals’ grains, the most important energy carriers are complex carbohydrates, with starch as the most commonly found polysaccharide [136]. A study published by Esteves et al. [159] indicates that lignin has a higher carbon content than polysaccharides, and as a result exhibits higher HHV values (MJ kg^−1^) compared with polysaccharides (18.6 MJ kg^−1^), which is the main factor behind the lower performance of cereal grain in terms of HHV performance compared with woody biomass. Various cereal grains can be in fact utilized as solid biofuels. For example, Keppel et al. [160] tested different species of cereals as a source of biomass for house heating—these included barley, triticale, wheat, and oats. Authors found that oats had superior combustion properties (19.28 MJ kg^−1^) compared with other cereals (approximately 18.2 MJ kg^−1^), with calorific values slightly lower than wood pellets (approximately 20 MJ kg^−1^) [161]. Oats (*Avena sativa*) can reach a calorific value ranging from approximately 15.4 to 18.7 MJ kg^−1^ [162]. Barley’s brewer’s spent grain’s (waste after the beer production process) HHV ranges from 17.3 MJ kg^−1^ to 17.5 MJ kg^−1^ [163]. Wheat grain’s HHV has been reported by Darguža and Gaile [161] to be at a level of approximately 16.5 MJ kg^−1^.

Rye grain has the potential to serve as renewable energy source in terms of its HHV. Moreover, the high content of non-structural carbohydrates make it a good raw material for bioethanol production [164]. However, timely agricultural practices, including harvest timing, are essential for maximizing fermentable sugar yields, with variations in crop maturity significantly affecting the bioethanol output [165].

Nevertheless, despite the promising HHV performance of rye grains, the idea of growing cereal grains for biofuels raises important questions about sustainability, especially when it comes to food security. Growing energy plants on agricultural land that is primarily devoted to food production can also be a subject of some moral dilemma. On the other hand, it seems reasonable to utilize grains with reduced quality characteristics for energy purposes. This includes kernels exhibiting inferior quality parameters immediately after harvest, as well as those that have been improperly stored (e.g., sprouted grain) or infested by some pests. Such conditions make grains unsuitable for consumption, prompting the search for alternative uses, such as for energy production [136]. Such approaches can build farms’ resilience by changing challenges (utilization of unsellable grains) into opportunity (energy source) [166]. As food demand around the world is growing, only lower-quality grains should be considered as potential biofuel or raw material for biofuel production. On the other hand, there is also a growing demand for renewable energy sources. This creates a unique opportunity for integrating the cereal grains of insufficient quality into biofuel value chains [167]. This aligns with recent efforts to enhance sustainability in both the food and energy sectors by utilizing waste streams [168]. Grains of cereals can be damaged by diseases and/or pests, which lowers their utilization potential as food (e.g., due to mycotoxin levels), but still can be potentially valuable in the biomass energy sector [169,170]. The energy obtained from low-quality cereal grains can contribute to farm resilience by decreasing the reliance of farms on external energy and thus increasing the energy autonomy of farms [171]. The energy can be used for heating households or farm buildings. Studies have shown that cereals, such as oats, yield substantial energy returns per unit mass when combusted, thus making them efficient biofuels [160,162].

## 5. Summary

Rye (*Secale cereale* L.) is a unique agricultural crop that can contribute significantly to both sustainable food systems and renewable energy production. Its ability to grow on poor soils and under challenging climatic conditions makes it a great crop in climate-resilient agriculture. The agronomic optimization of rye’s grain quality through genotype selection, crop rotation, soil management, and fertilization improves its technological and nutritional properties but also supports food safety.

Rye’s grain has a high fiber and bioactive compound content, including phenolic acids, lignans, alkylresorcinols, and benzoxazinoids. The presence of these substances means that rye possesses some antioxidant, anti-inflammatory, and disease-preventing properties, which can contribute to healthy, dietary diversification.

The use of low-quality, sprouted, pest-infested, or otherwise unsuitable for human consumption grains offers a practical solution to enhance farm energy autonomy while addressing food versus fuel debates. Utilizing such grains at the farm level for energy purposes aligns with the circular economy principles and strengthens farm resilience by turning agricultural losses into renewable energy opportunities. Despite these advantages, it seems advisable to balance the use of food crops for energy production with global food security concerns. Current strategies encourage waste-stream integration and utilization, with non-marketable biomass (as waste grains) a promising alternative to build the environmental and socio-economic benefits of cereal-based bioenergy systems.

New breeding programs for rye are needed to fully utilize its multi-dimensional potential. Despite its high agronomic and health-promoting advantages, the improvement of its grain quality, especially in terms of the fiber and micronutrient content, might strengthen rye’s importance in the agricultural and food sectors. The selective breeding of rye in order to achieve a higher content of phenols, lignans, and benzoxazinoids also has the potential to make rye an important bio-functional food source.

## Figures and Tables

**Table 1:** Content of selected components in rye whole grains and light, low-extract flours.

Component	Whole Grains	Flour	References
Aleuren fraction	11–14%	<0.1%	[98,99]
Embryo	3%	<0.1%	[98]
Total fiber	13.0–22.2%	3%	[98,100]
Insoluble fiber	11.5%	1.90%	[98]
Soluble fiber	1%	1.00%	[98]
Protein	8–14%	14%	[98,101]
Fat	1.5–3%	1.40%	[98,102]
Starch and other sugars	70%	83%	[98]
Minerals	2%	0.6%	[98]
Zinc (µg g^−1^)	29	8	[98]
Iron (µg g^−1^)	35	13	[98]
Selenium (µg g^−1^)	1.4–7	0.02	[98,103,104]
B-tocotrienol (µg g^−1^)	33	5.7	[98]
Vitamin B6 (mg g^−1^)	8	1.4	[98]
Folic acid (mg g^−1^)	23–140	0.11	[98,105,106]
Feluric acid (mg g^−1^)	5	0.4	[99]
Phosphorus (mg g^−1^)	1.8–4.2	0.1	[91,107]

Source: [98,99,100,101,102,103,104,105,106,107].

## Data Availability

The data presented in this study are available on request from the corresponding author. The data are not publicly available due to the internal rules of IUNG-PIB (the data owner).
